# Extracorporeal Membrane Oxygenation for Pneumocystis Pneumonia: Outcomes in Patients From the Extracorporeal Life Support Organization

**DOI:** 10.1097/CCE.0000000000001414

**Published:** 2026-05-05

**Authors:** Kyle J. Henry, Emily R. Thompson, Kealy Ham, Holenarasipur R. Vikram, Rodrigo Cartin-Ceba, Georges Khattar, Jonathan D’Cunha, Andrew H. Limper, Ayan Sen, Bhavesh Patel, Aaron M. Pulsipher

**Affiliations:** 1 Department of Critical Care, Mayo Clinic, Phoenix, AZ.; 2 Division of Clinical Trials and Biostatistics, Mayo Clinic Arizona, Phoenix, AZ.; 3 Division of Infectious Diseases and Transplant Center, Mayo Clinic, Phoenix, AZ.; 4 Division of Cardiothoracic Surgery, Mayo Clinic, Phoenix, AZ.; 5 Division of Pulmonary and Critical Care, Mayo Clinic, Rochester, MN.

**Keywords:** acute respiratory distress syndrome, immunocompromised host, *Pneumocystis jirovecii* pneumonia, venovenous extracorporeal membrane oxygenation

## Abstract

**OBJECTIVES:**

*Pneumocystis jirovecii* pneumonia (PCP) can cause severe hypoxemic respiratory failure in immunocompromised patients. Contemporary outcomes of venovenous extracorporeal membrane oxygenation (VV ECMO) support for PCP are poorly characterized, and existing reports are limited to small case series.

**DESIGN:**

Retrospective cohort study.

**SETTING:**

International registry study using the Extracorporeal Life Support Organization (ELSO) registry.

**PATIENTS/SUBJECTS:**

Adults with PCP-associated hypoxemic respiratory failure supported with VV ECMO between 2011 and 2024.

**INTERVENTIONS:**

VV ECMO.

**MEASUREMENTS AND MAIN RESULTS:**

A total of 209 patients with PCP-associated respiratory failure supported with VV ECMO were identified. The overall in-hospital mortality was 60.8%. Survivors were younger than nonsurvivors (median age 41.9 vs. 46.8 yr; *p* = 0.047). Duration of mechanical ventilation before VV ECMO was longer among nonsurvivors (median 4.7 vs. 1.6 d; *p* = 0.019). The proportion of patients with HIV infection was similar among survivors and nonsurvivors (19.5% vs. 22.1%; *p* = 0.73). Pre-ECMO vasopressor use, prone positioning, and renal replacement therapy were common and did not differ between groups. Clinical course was characterized by prolonged VV ECMO support (median 18.6 d) and frequent complications, including pneumothorax (21.1%), renal replacement therapy during VV ECMO (31.6%), intracranial hemorrhage or stroke (7.7%), and major pulmonary or gastrointestinal hemorrhage (16.8%). PCP accounted for a small proportion of VV ECMO runs reported to the ELSO registry throughout the study period at 0.36% of reported cases. No significant temporal trend in mortality was observed.

**CONCLUSIONS:**

PCP-associated respiratory failure supported with VV ECMO is associated with substantial mortality and prolonged VV ECMO support. These findings provide contemporary benchmarks that may inform VV ECMO candidacy discussions and expectations in this challenging patient population.

**KEY POINTS**

**Question**: What are the contemporary outcomes of adults with *Pneumocystis jirovecii* pneumonia (PCP)-associated hypoxemic respiratory failure supported with venovenous extracorporeal membrane oxygenation (VV ECMO)?

**Findings**: In this retrospective cohort study of 209 adults in the Extracorporeal Life Support Organization registry supported with VV ECMO for PCP between 2011 and 2024, in-hospital mortality was 60.8%, and VV ECMO support was frequently prolonged. Baseline HIV prevalence was similar among survivors and nonsurvivors, and PCP represented less than 1% of all VV ECMO runs reported to the registry.

**Meaning**: PCP-associated respiratory failure supported with VV ECMO is rare but associated with substantial mortality and prolonged support, providing important benchmarks to inform VV ECMO candidacy discussions and expectations.

*Pneumocystis jirovecii* pneumonia (PCP; also termed PJP) is a life-threatening opportunistic fungal infection that affects immunocompromised individuals. Although historically associated with advanced HIV/AIDS, the incidence in non-HIV immunocompromised individuals now exceeds that of people with HIV in high-income nations. Non-HIV patients often present with more fulminant disease and experience higher mortality with reported rates of 41–67% among critically ill cohorts (1–3).

Venovenous extracorporeal membrane oxygenation (VV ECMO) is recommended for patients with acute respiratory failure that has been refractory to optimal conventional therapy, when recovery is expected or as a bridge to lung transplantation (4–6). Despite this, immunosuppression has long been considered a relative contraindication to VV ECMO, and current American Thoracic Society and International Society for Heart and Lung Transplantation guidelines continue to list major pharmacologic immunosuppression as such (4, 7). This historical caution has contributed to hesitancy in offering VV ECMO support to patients with PCP.

Despite the severity of PCP-associated respiratory failure, outcomes among patients supported with VV ECMO are poorly defined, with existing data limited to small, heterogeneous case series. In our recent multicenter analysis, seven of ten PCP patients supported with VV ECMO survived—including several without HIV infection—suggesting that VV ECMO may offer benefit in carefully selected patients (8). Conversely, Rilinger et al (9) reported an overall VV ECMO survival of only 22.2% in a PCP cohort and demonstrated substantially higher survival in patients with HIV compared with non-HIV immunosuppression, underscoring important differences in underlying host biology. Collectively, these observations emphasize both the substantial mortality risk and the limited, inconsistent evidence base guiding VV ECMO use in PCP.

To better define contemporary outcomes, we analyzed data from the Extracorporeal Life Support Organization (ELSO) registry, which captures international, real-world VV ECMO use across diverse centers. We hypothesized that outcomes in this large multicenter cohort could help provide greater clarity regarding the role of VV ECMO in the supportive management of severe PCP-associated respiratory failure.

## MATERIALS AND METHODS

We conducted a retrospective cohort study using data from the ELSO registry. Because this study used de-identified data, The Mayo Clinic Institutional Review Board (IRB) determined through the institutional Human Subjects Research determination process that the activity did not require IRB review. Adult patients (≥ 18 yr) supported with VV ECMO between January 1, 2011, and December 31, 2024, were included if they had a recorded microbiologic diagnosis of PCP. For each patient, we extracted demographic characteristics and pre-ECMO treatment variables including vasopressor use, neuromuscular blockade, prone positioning, pulmonary vasodilator use, and duration of mechanical ventilation before VV ECMO cannulation. HIV infection status was recorded.

Clinical course variables included duration of VV ECMO support and timing of PCP diagnosis relative to VV ECMO initiation. Complications assessed included pneumothorax, renal replacement therapy during VV ECMO, ischemic stroke or intracranial hemorrhage, and pulmonary or gastrointestinal hemorrhage. Discharge disposition, including survival to hospital discharge and discharge location, was also collected.

Categorical variables are presented as counts and percentages, and continuous variables as medians with interquartile ranges (IQRs). Baseline characteristics were compared between survivors and nonsurvivors using the Wilcoxon rank-sum test for continuous variables and Fisher exact tests for categorical variables. Clinical course and outcome variables were summarized descriptively. Missing data were not imputed, and analyses were performed using available-case (complete-case) data for each variable.

Annual PCP case frequency was indexed to the total number of VV ECMO runs reported to the ELSO registry for each year. Temporal trends in the proportion of PCP-associated VV ECMO cases were evaluated using linear regression with calendar year modeled as a continuous variable. Annual mortality rates were calculated and temporal trends assessed using binomial regression. Statistical analyses were performed using Stata, Version 19.5 (StataCorp, College Station, TX), with two-sided significance defined as *p* value of less than 0.05.

## RESULTS

A total of 209 adult patients with PCP-associated respiratory failure supported with VV ECMO between 2011 and 2024 were identified in the ELSO registry. One patient with PCP identified after the VV ECMO course was excluded. Baseline characteristics stratified by survival are shown in **Table 1**. Survivors were younger than nonsurvivors (41.9 yr [IQR, 30.4–53.7 yr] vs. 46.8 yr [IQR, 35.4–57.1 yr]; *p* = 0.047). Sex distribution was similar between groups (male 61.0% vs. 68.5%; *p* = 0.25), as were body mass index values (25.7 [IQR, 22.2–30.3] vs. 25.0 [IQR, 21.5–30.7]; *p* = 0.52). HIV infection was present in 19.5% of survivors and 22.1% of nonsurvivors (*p* = 0.73).

**TABLE 1.** Baseline Demographics and Pre-Extracorporeal Membrane Oxygenation Support

Variable	Survivor (*n* = 82)	Nonsurvivor (*n* = 127)	*p*
Demographics			
Age, yr	41.9 (30.4–53.7)	46.8 (35.4–57.1)	0.047
Body mass index^a^	25.7 (22.2–30.3)	25.0 (21.5–30.7)	0.52
Sex (male)^b^	51 (61.0%)	87 (68.5%)	0.25
Pre-ECMO clinical characteristics			
HIV	16 (19.5%)	28 (22.1%)	0.73
Inotropy	4 (4.9%)	5 (3.9%)	0.74
Vasopressors	46 (56.1%)	78 (61.4%)	0.47
Paralysis	49 (59.8%)	83 (65.4%)	0.46
Prone positioning	20 (24.4%)	30 (23.6%)	1.00
Pulmonary vasodilator	20 (24.4%)	37 (29.1%)	0.53
Mechanical ventilation duration before ECMO, d^c^	1.6 (0.8–5.5)	4.7 (1.1–7.2)	0.019
Renal replacement therapy	9 (11.0%)	11 (8.7%)	0.63
*Pneumocystis jirovecii* pneumonia diagnosis before ECMO cannulation	37 (45.1%)	72 (56.7%)	0.12

ECMO = extracorporeal membrane oxygenation.

a Body mass index was missing in 14 survivors and 36 nonsurvivors.

b One patient with unknown biological sex.

c Mechanical ventilation duration was missing in ten survivors and 15 nonsurvivors.

Data are presented as median (interquartile range) or *n* (%).

Vasopressor and pulmonary vasodilator use was similar between groups as was the use of prone positioning. Duration of mechanical ventilation before VV ECMO initiation was longer among nonsurvivors (4.7 d [IQR, 1.1–7.2 d] vs. 1.6 d [IQR, 0.8–5.5 d]; *p* = 0.019). PCP diagnosis was established before VV ECMO cannulation in 45.1% of survivors and 56.7% of nonsurvivors (*p* = 0.12).

Clinical course and outcomes are summarized in **Table 2**. Overall in-hospital mortality was 60.8% (127/209). VV ECMO runs were prolonged, with a median duration of 18.6 days (IQR, 9.6–34.0 d) across the cohort. Complications were common. Pneumothorax occurred in 21.1% of patients, renal replacement therapy during VV ECMO in 31.6%, and major pulmonary or gastrointestinal hemorrhage in 16.8%. Neurologic complications including ischemic stroke or intracranial hemorrhage occurred in 7.7% of patients.

**TABLE 2.** Clinical Course, Complications, Coinfections, and Outcomes

Variable	Survivor (*n* = 82)	Nonsurvivor (*n* = 127)	Total (*n* = 209)
Coinfections			
Cytomegalovirus DNAemia	13 (15.9%)	21 (16.5%)	34 (16.3%)
Pulmonary *Aspergillus* isolation	3 (3.7%)	13 (10.2%)	16 (7.7%)
Complications			
Pneumothorax	15 (18.3%)	29 (22.8%)	44 (21.1%)
Renal replacement therapy during ECMO	13 (15.9%)	53 (41.7%)	66 (31.6%)
Stroke (ischemic or hemorrhagic)	0 (0%)	16 (12.6%)	16 (7.7%)
Pulmonary or gastrointestinal hemorrhage	9 (11.0%)	26 (20.5%)	35 (16.8%)
Outcomes			
ECMO duration, d	14.8 (8.8–32.8)	21.0 (9.8–36.3)	18.6 (9.6–34.0)
Hospital length of stay, d	42.5 (29–67)	28 (16–46.5)	34.5 (18–52)
Disposition among survivors^a^			
Home	25 (30.5%)		
Transferred to another hospital	31 (37.8%)		
Long-term acute care/rehabilitation	21 (25.6%)		
Other, unknown	5 (6.1%)		

ECMO = extracorporeal membrane oxygenation.

a Three patients were transferred to another hospital while still receiving ECMO support.

Data are presented as median (interquartile range) or *n* (%).

Among survivors, hospital length of stay was 42.5 days (IQR, 29–67 d). Of survivors discharged from the hospital, 30.5% were discharged home, 37.8% were transferred to another hospital, and 25.6% required long-term acute care or rehabilitation facilities.

Between 2011 and 2024, PCP-associated respiratory failure accounted for a small proportion of VV ECMO runs reported to the ELSO registry, consistently representing less than 1% of all VV ECMO cases as shown in **Supplemental Figure 1** (https://links.lww.com/CCX/B625) with no significant temporal trend (*p* = 0.35).There was no significant temporal trend in mortality over the study period (*p* = 0.14).

## DISCUSSION

In this large, contemporary cohort of adults with PCP supported with VV ECMO in the ELSO registry, in-hospital mortality was 60.8%. Survivors were younger and had fewer days of mechanical ventilation before VV ECMO cannulation than nonsurvivors. VV ECMO support and hospital length of stay were prolonged, and complications were common during the clinical course.

PCP-related respiratory failure was an uncommon indication for VV ECMO, consistently representing less than 1% of all VV ECMO runs reported in the ELSO registry. Although the annual number of cases increased over time, the overall rarity of PCP supported with VV ECMO likely contributes to the limited and inconsistent evidence base describing VV ECMO outcomes. Prior reports have varied substantially: Rilinger et al (9) described a 22% survival rate in a multicenter cohort, whereas Pulsipher et al (8) reported 70% survival in a multicenter case series. Another study evaluating fungal causes of acute respiratory distress syndrome (ARDS) reported an overall survival rate of 73%, including 80% survival among patients with PCP (10). The survival rate observed in the present study—roughly 39%—lies between these estimates and likely reflects a more heterogeneous case mix and center distribution than in previous reports. Notably, survival in this cohort remains lower than typical outcomes for VV ECMO in broader ARDS populations, where survival commonly ranges from 55% to 65% (11, 12) In this context, VV ECMO in PCP-related respiratory failure may be best viewed as a supportive therapy serving as a trial to recovery, understanding the prolonged course and high likelihood of mortality.

An important observation in this cohort is that nearly half of patients were cannulated for VV ECMO before a diagnosis of PCP was established. This finding highlights the diagnostic uncertainty that frequently accompanies severe ARDS, particularly in immunocompromised patients where the differential diagnosis is broad and microbiologic confirmation may require invasive testing or specialized assays. In many cases, the decision to initiate VV ECMO must be made before the underlying etiology of respiratory failure is fully defined. This underscores the importance of maintaining a broad diagnostic evaluation in patients with severe ARDS and immunocompromise, including consideration of opportunistic infections such as PCP even when the diagnosis is not immediately apparent.

This study has limitations inherent to retrospective analyses of registry data. ELSO reporting is voluntary and may be subject to misclassification, particularly regarding immunosuppression type and severity. The registry also does not allow confirmation of PCP pathogenicity; diagnostic methods are not captured, and PCP may represent colonization, particularly in non-HIV hosts. Center-level practice variation in VV ECMO initiation, cannulation, and management could not be assessed and likely contributed to outcome variability. Further, the patient population is restricted to ELSO-reporting centers and may lack generalizability. Finally, there is no comparison cohort of patients who were not deemed VV ECMO candidates to further clarify VV ECMO candidacy decisions.

## CONCLUSIONS

In this international registry cohort, PCP-associated respiratory failure supported with VV ECMO was associated with substantial mortality and prolonged VV ECMO support. Survivors were younger and had shorter durations of mechanical ventilation before VV ECMO cannulation, while complications during the VV ECMO course were common. These findings provide contemporary descriptive benchmarks for centers considering VV ECMO in patients with severe PCP-related respiratory failure and highlight the need for counseling regarding expected clinical courses and outcomes.

## Supplementary Material
