# Supplementary material for: Extracorporeal Membrane Oxygenation for Pneumocystis Pneumonia: Outcomes in Patients From the Extracorporeal Life Support Organization
Source: Crit Care Explor. 2026 May 5;8(5):e1414. doi: 10.1097/CCE.0000000000001414 (PMC13143505; doi:10.1097/CCE.0000000000001414)
Supplement: Supplementary file 1 [file cc9-8-e1414-s001.pdf]

Extracorporeal Membrane Oxygenation for Pneumocystis Pneumonia: Outcomes in Patients From the Extracorporeal Life Support Organization Supplemental Data

Page Number

Supplemental Figure 1……………………………………………………………..……2

Supplemental Figure 1 Legend………………………………………………………….2

Supplemental Figure 1

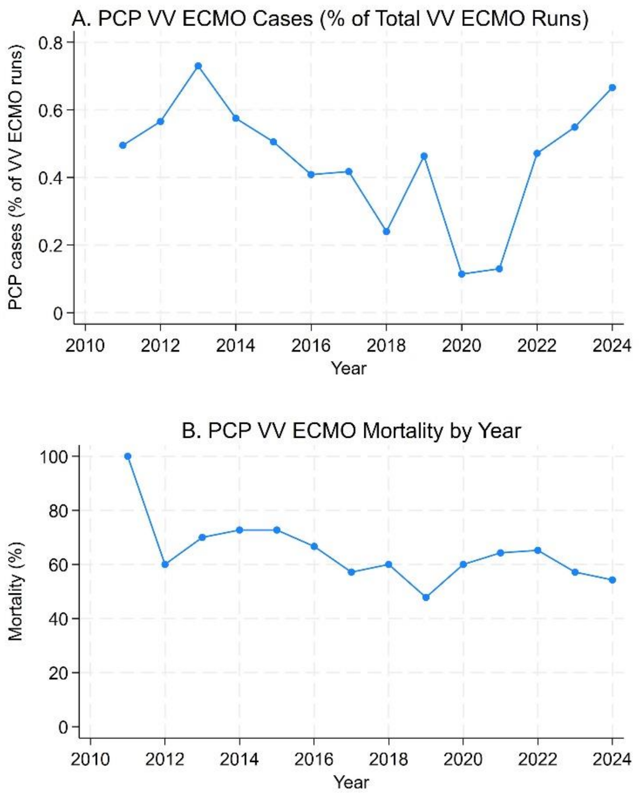

Supplemental Figure 1 Legend

Panel A shows the annual proportion of Pneumocystis jirovecii pneumonia (PCP) cases among all adult venovenous extracorporeal membrane oxygenation (VV ECMO) runs reported to the Extracorporeal Life Support Organization (ELSO) registry from 2011 through 2024. Panel B shows yearly in-hospital mortality among patients with PCP supported with VV ECMO. Temporal trends were evaluated using linear regression for annual case proportions and binomial regression for yearly mortality rates. No statistically significant temporal trends were observed.
